# Primary neuroendocrine tumour of the breast: a case report and review of the literature

**DOI:** 10.3332/ecancer.2015.607

**Published:** 2015-12-22

**Authors:** Sara Tato-Varela, Rosa Albalat-Fernández, Sara Pabón-Fernández, Enrique Rodríguez Zarco, Manolo La Calle-Marcos

**Affiliations:** 1 Clinical Management Unit, Gynaecology and Obstetrics, Hospital Universitario Virgen Macarena, Sevilla 41008, Spain; 2 Pathological Anatomy Service, Hospital Universitario Virgen Macarena, Sevilla 41008, Spain

**Keywords:** neuroendocrine carcinoma, endocrine carcinoma, invasive carcinoma, breast

## Abstract

Primary neuroendocrine tumour of the breast is a rare entity that first appeared in the 2003 World Health Organisation (WHO) classification of breast tumours. The data currently available on its prognosis are contradictory, although it seems clear that histological varieties such as small cell neuroendocrine carcinoma have a worse prognosis, due to their low degree of differentiation. The treatment of choice is surgery, and the indications for chemotherapy or radiotherapy do not differ greatly from those used for other breast tumours. It is crucial to underline the difficulty of establishing treatment protocols due to the low incidence of this histological type.

## Introduction

Malignant neuroendocrine tumours of the breast are infrequently occurring growths that account for less than 0.1% of all breast cancers and less than 1% of all neuroendocrine tumours [1]. It is more common for this kind of tumour to originate in the gastrointestinal tract or in the bronchopulmonary system, where the global prevalence is one to two cases per 100,000 people [2]. Global survival rates for neuroendocrine breast cancers stand at 80% after five years, depending in each case on the histological grade and the anatomo-clinical stage [3]. The prognosis improves in cases of primary neuroendocrine tumours that are small in size, have no axillary involvement, and where hormonal receptors are positive [4]. Below, we present a clinical case study of a neuroendocrine breast cancer that began with axillary metastasis, together with a review of the literature related to this infrequent histological type.

## Clinical case study

**Figure 1.**
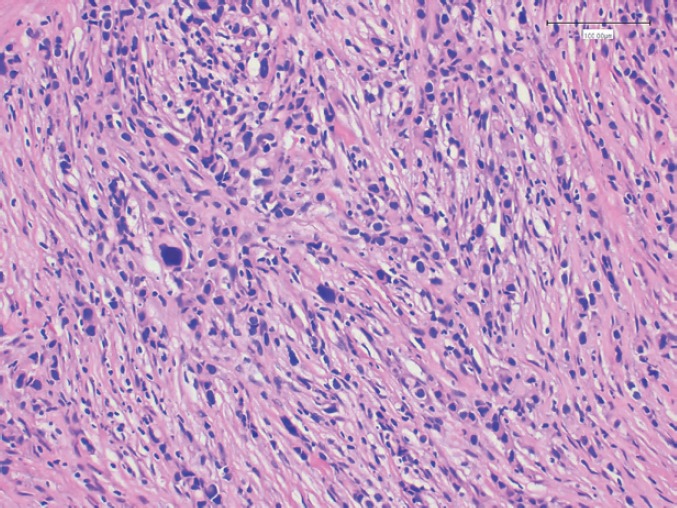
Tumour staining with hematoxylin and eosin.

**Figure 2.**
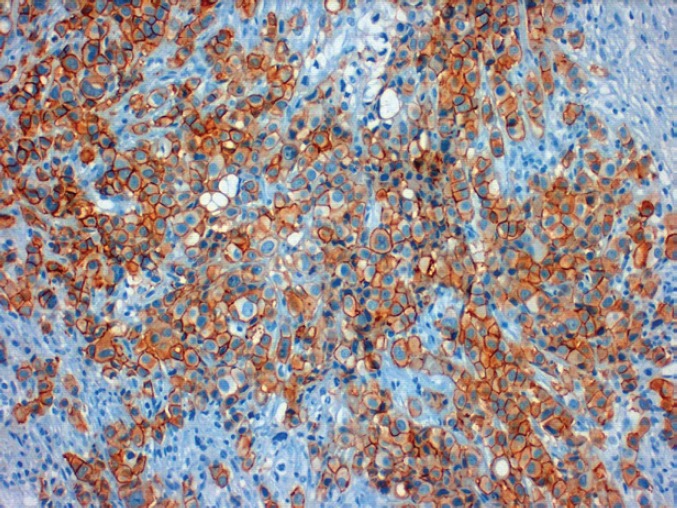
Positive tumour staining for HER-2 (3+).

**Figure 3.**
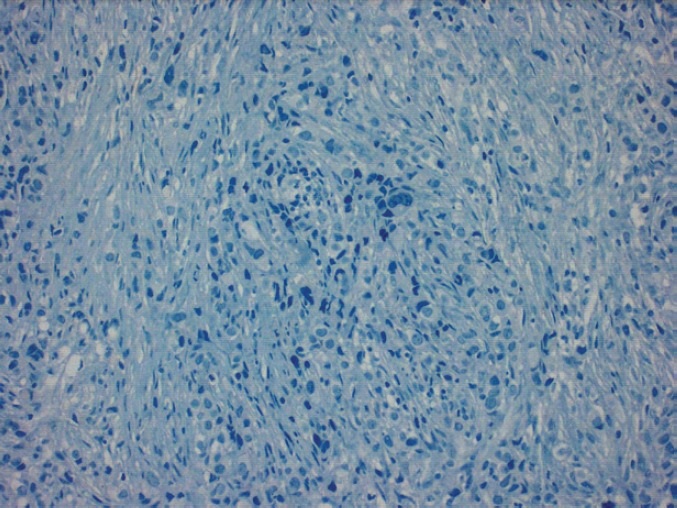
Absence of E-cadherin staining observed.

**Figure 4.**
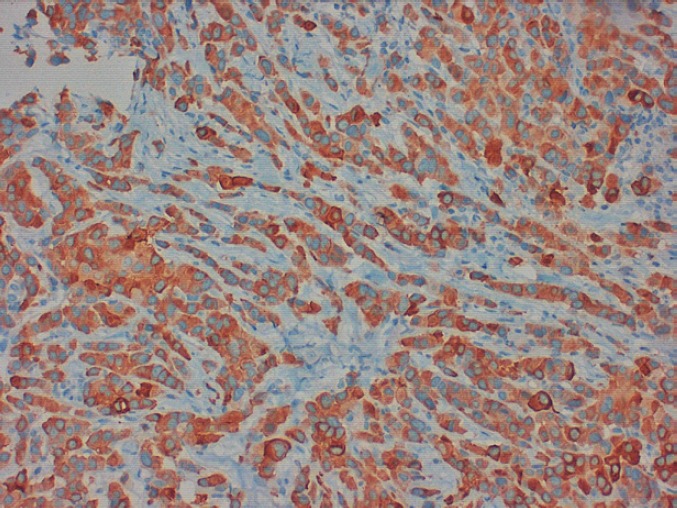
Positive staining for synaptophysin in more than 50% of the tissue permits the diagnosis of a neuroendocrine tumour.

In 2013, the patient, a 62-year-old woman, consulted her primary care physician regarding a painful axillary lymph node that had recently appeared. She had no medical history of interest other than a laparoscopic cholecystectomy and grade 1 obesity. Palpation identified a 2 cm nodule in the inferior outer quadrant of the left breast and a 3 cm nodule in the ipsilateral axilla. The patient underwent a mammogram which showed increased trabeculation in the upper quadrants and irregular densities in the axillary region. The subsequent ultrasound showed a 3 cm irregular lesion in the inferior outer quadrant of the left breast (axillary region), which had multiple satellite lymphadenopathies, the largest of which was 2 cm. With a diagnosis highly suggestive of malignancy (BIRADS V), the patient underwent a core needle biopsy (CNB) on the breast nodule and an axillary fine-needle aspiration biopsy (FNAB). The breast CNB yielded the diagnosis of invasive pleomorphic carcinoma (Figure 1) with positive hormonal receptors (oestrogens 100% and progesterone 80%, Ki67 70%, HER-2 positive (3+) (Figure 2), CK19 positive, E-cadherin negative (Figure 3), and P63 negative. The positivity of synaptophysin (Figure 4) suggested that the tumour was neuroendocrine in origin. The axillary FNAB showed metastasis of the carcinoma.

After taking her case to committee for study, the decision was made to start treatment with adjuvant chemotherapy and thereafter to consider surgery based on the outcome. Staging image tests were carried out on the breast lesion along with implantation of metal markers. Nuclear magnetic resonance imaging (NMRI) showed involvement of the pectoral fascia, 2 cm in length while computerised tomography (CT) showed the presence of suspicious supraclavicular adenopathies of also 2 cm. The definitive staging was T2 N+ M0.

A month after diagnosis, the patient began chemotherapy treatment involving docetaxel, trastuzumab, and carboplatin. The decision was made to administer six cycles of treatment, one every 21 days. The first cycle involved 80 mg/m^2^ of docetaxel, 8 mg/kg of trastuzumab, and 6 UI of carboplatin, while those that followed involved lower doses (60 mg/m^2^ of docetaxel, 6 mg/kg of trastuzumab, and 4 UI of carboplatin). The patient required the administration of granulocyte colony stimulating factor and presented a good tolerance of the chemotherapy, experiencing only light diarrhoea after the first three cycles. The breast examination after completion of the six cycles of chemotherapy showed a notable reduction in the previously described lesions, a finding that was confirmed via NMRI, which showed a 10 mm lesion of the left breast and a 12 mm ipsilateral axillary lesion with no surrounding adenopathy.

The patient underwent surgery six months after the initial diagnosis, when a left mastectomy was performed along with an ipsilateral axillary lymphadenectomy. There were no postoperative incidents and pathological anatomy reported the absence of residual neoplasia (grade V on the Miller– Payne scale) and fibrosis. After surgery, the decision was made to administer adjuvant chemotherapy treatment (trastuzumab 6 mg/kg every 21 days for a year), hormone therapy with letrozole, and radiotherapy (40.05 Gray (Gy)) on left breast volume, with fractionation of 2.67 Gy/fraction and irradiation of the left supraclavicular region. The level III axillary treated with radiotherapy up to 40.14 Gy with fractionation of 2.23/fraction). The patient tolerated the adjuvant treatment adequately, presenting with just slight anteroseptal hypokinesis at the cardiac level which did not require treatment, and a grade 1 radiodermatitis in skin folds. Of note at the time of writing this article, she was asymptomatic.

## Discussion

Neuroendocrine breast tumours are extremely rare and may be both primary lesions and metastatic lesions. They derive from neuroendocrine cells that are present throughout the body, the most frequent primary localisations being the bronchopulmonary and digestive tracts (especially the small intestine) [5]. In 2003, the WHO first recognised the breast localisation as a differentiated entity that presents morphological tumour characteristics similar to those of neuroendocrine tumours in other localisations, with expression of neuroendocrine markers in more than 50% of the cell population [6]. Although it can be very difficult, it is vital to differentiate between the primary or metastatic origin of the neuroendocrine tumour in the breast as each requires a different therapeutic approach [5]. In general metastases to the breast are frequent tumours, accounting for 2% of breast tumours, whose most common localisations are melanoma in adults and rhabdomyosarcoma in children. In the case of neuroendocrine tumours, the most frequent primary localisations are the ilium followed by the appendix, the duodenum, the pancreas, and the lung [2]. Primary neuroendocrine cancer of the breast generally presents in mature women (between the sixth and seventh decade of life) and its incidence increases with age; this is not the case for metastatic neuroendocrine tumours, which appear an average of ten years before the primary tumours [7].

The diagnosis of a primary neuroendocrine tumour is always a diagnosis of exclusion [3], and it may be carried out as long as there is a component of ductal carcinoma *in situ* in the sample, and that the existence of tumours in other localisations has been ruled out [2]. The differentiation between a primary and metastatic neuroendocrine cancer of the breast is impossible to achieve based solely on mammogram or breast ultrasound [2]. Multiple imaging tests may be used to help localise the primary tumour. CT is the most commonly used test, since it enables a broad field of vision (pelvis, abdomen, and thorax) as well as a suitable level of detail of the vascular anatomy, although it has little sensitivity when it comes to detecting small hepatic lesions. NMRI is a powerful tool for the evaluation of neuroendocrine tumours, especially for visualising hepatic involvement. Acquisitions of multi-sequence images associated with the introduction of hepatocyte-specific contrast agent such as gadoxetic acid make it possible to measure lesions with a high level of precision. Ultrasound may be used both to guide the biopsy and diagnose hepatic lesions as well as to rule out secondary cardiac involvement in carcinoid syndrome prior to performing an operation. Endoscopy makes it possible to evaluate gastric, ileal, colonic, and rectal lesions directly. Functional imaging tests such as OctreoScan among others may prove useful in the evaluation of metastatic disease, or to decide the extent of the resection to be carried out prior to the operation. Moreover, multiple urinary and blood biomarkers have been described, such as chromogranin-A and pancreastatin, whose use as a complement to imaging tests can be useful when it comes to determining the localisation of the primary cancer, although the definitive diagnosis will always be carried out after the histological study of the sample or biopsy is obtained [8].

Neuroendocrine tumours of the breast lack a specific clinic [9]: for the most part, patients consult physicians about a breast nodule, either in isolation or in association with other symptoms, such as a well-defined cutaneous erythematous or purple plaque [3]. Carcinoid syndrome (including diarrhoea, flushing, and bronchospasm) can appear in 5–10% of patients with a neuroendocrine tumour [2].

Radiological diagnostics do not present any distinctive characteristics [9], Although frequent occurrence of masses with speculated or lobulated margins have been described in mammograms as well as microlobulated and homogeneously hypoechoic masses in breast ultrasound [10]. It is worth highlighting that in none of the cases described to date has posterior acoustic shadowing or a cystic component been detected in the ultrasound, although [11] none of the previously described characteristics represent a specific finding. On carrying out a biopsy, we must be very careful with regard to hormonally active tumours as we may trigger a carcinoid crisis with the puncture [2].

Given the low incidence of neuroendocrine breast cancer, the optimal treatment strategy remains controversial [12]. It seems clear that the principal treatment is surgery [1] and that the primary breast tumours must be dealt with in a similar way to ductal carcinoma of the breast. In the case of metastatic tumours, a lumpectomy is recommended to reduce the size of the tumour mass, avoiding mastectomy or axillary dissection [5]. The benefits of adjuvant therapy have not been demonstrated in the literature because of absence of clinical trials and the low incidence of the disease [7]. Inspite of this, it is administered following the same criteria as that for other breast tumours [3].

Once the sample has been removed, the microscopic examination shows alveolar structures or solid sheets of cells with a tendency to produce peripheral palisades [13]. According to the WHO classification, the diagnosis requires immunohistochemical expression of synaptophysin or chromogranin in more than 50% of the cells [4]. Positivity to hormonal receptors is a practically constant characteristic, whereas the expression of c-erbB-2 is less frequent, appearing in two of the 22 cases reported to date [11]. Expression of the oncoprotein HER-2 not only plays an important role in the prognosis and the nodal extension of the tumour, but a study carried out by Horiguchi and colleagues demonstrates that 80% of tumours with positive cytoplasmic staining for this marker also expressed chromogranin A or specific neuronal enolase–in other words they display neuroendocrine differentiation [14]. At the cytogenetic level, it seems that neuroendocrine tumours of the breast share more similarities with primary breast tumours than that with neuroendocrine tumours in other localisations, although they do share cytogenetic abnormalities with them, such as trisomy 7 and 12 [15]. Depending on the degree of differentiation and cellular morphology, neuroendocrine cancer of the breast may be classified into one of the following histopathological subtypes: solid carcinoma, oat cell, large cell [12], and atypical [3].

The prognosis for neuroendocrine cancer of the breast is controversial, although it is a slow-developing tumour [3] and it seems that global disease-free survival is greater in patients with primary neuroendocrine tumours of the breast than in patients with other breast tumours or other neuroendocrine tumours [12]. Its development essentially depends on the anatomo-clinical stage and the histological grade [3] (excepting small cell or oat cell carcinomas, 45% of neuroendocrine tumours of the breast are well differentiated, and 40% are moderately differentiated [12]). Other prognostic factors include mucinous differentiation, expression of hormonal receptors [4], HER-2 positivity [12], patient age, tumour markers, and tumour secretion [3].

## Conclusion

Primary neuroendocrine tumours of the breast are infrequent tumours that appear in women aged 60–70 years. Their diagnosis is complex as they lack defining clinical and radiological characteristics and also given the fact that their low incidence makes it impossible to establish an optimal treatment protocol. In general, it is recommended to follow a treatment similar to that used for ductal carcinoma of the breast. Diagnosis is achieved via staining with neuroendocrine markers. Although survival depends on factors such as tumour differentiation and stage, it is found to be in excess of 80% after five years.

## Conflicts of interest

The authors declare that there are no conflicts of interest.

## Authors’ contributions

All the authors participated in the bibliography search. STV was in charge of producing the text. All the authors approved the final version of the document.
